# Isotropic plasticity of β-type Ti-29Nb-13Ta-4.6Zr alloy single crystals for the development of single crystalline β-Ti implants

**DOI:** 10.1038/srep29779

**Published:** 2016-07-15

**Authors:** Koji Hagihara, Takayoshi Nakano, Hideaki Maki, Yukichi Umakoshi, Mitsuo Niinomi

**Affiliations:** 1 Department of Adaptive Machine Systems, Graduate School of Engineering, Osaka University, 2-1 Yamadaoka, Suita, Osaka 565-0871, Japan; 2 Division of Materials and Manufacturing Science, Graduate School of Engineering, Osaka University, 2-1 Yamadaoka, Suita, Osaka 565-0871, Japan; 3 Institute for Materials Research, Tohoku University, 2-1-1 Aoba-ku, Sendai, Miyagi 980-8577, Japan

## Abstract

β-type Ti-29Nb-13Ta-4.6Zr alloy is a promising novel material for biomedical applications. We have proposed a ‘single crystalline β-Ti implant’ as new hard tissue replacements for suppressing the stress shielding by achieving a drastic reduction in the Young’s modulus. To develop this, the orientation dependence of the plastic deformation behavior of the Ti-29Nb-13Ta-4.6Zr single crystal was first clarified. Dislocation slip with a Burgers vector parallel to <111> was the predominant deformation mode in the wide loading orientation. The orientation dependence of the yield stress due to <111> dislocations was small, in contrast to other β-Ti alloys. In addition, {332} twin was found to be operative at the loading orientation around [001]. The asymmetric features of the {332} twin formation depending on the loading orientation could be roughly anticipated by their Schmid factors. However, the critical resolved shear stress for the {332} twins appeared to show orientation dependence. The simultaneous operation of <111> slip and {332} twin were found to be the origin of the good mechanical properties with excellent strength and ductility. It was clarified that the Ti-29Nb-13Ta-4.6Zr alloy single crystal shows the “plastically almost-isotropic and elastically highly-anisotropic” nature, that is desirable for the development of ‘single crystalline β-Ti implant’.

Ti alloys are some of the most attractive and widely used biomedical implant materials^1^,^2^. They are generally preferred to 316L stainless steel and Co-Cr-Mo alloys owing to their low weight, excellent biocompatibility, corrosion resistance, high specific strength, and low elastic modulus^3^. Among these properties, control of the Young’s modulus has been important in the recent development of implant materials. A reduction in Young’s modulus is essential to prevent bone degradation and bone resorption, caused by the difference in the Young’s moduli between a bone replacement material and natural human bone, i.e. the stress shielding by the implant^4^. Among the Ti alloys, the Ti-6Al-4V alloy with an α + β two-phase microstructure is currently widely used as an implant material. The Young’s modulus of the Ti-6Al-4V alloy is ~110 GPa, approximately half of that of 316 stainless steel or Co-Cr-Mo alloys^2^,^3^. However, the Young’s modulus is still significantly higher than that of the cortical bone in humans (~10–30 GPa)^5^. In addition, studies considering the long-term health problems caused by Al and V ion release from Ti-6Al-4V have been reported^6^. Thus, novel Ti alloys with lower Young’s moduli and improved biocompatibility are required. Recently, β(bcc)-type Ti-Nb-Ta-Zr quaternary alloys containing only non-toxic bcc-stabilized elements (Nb and Ta) have received growing interest^7^,^8^,^9^,^10^,^11^,^12^, as β-Ti alloys generally exhibit lower Young’s moduli than α-Ti alloys, and their processabilities are also better owing to their higher crystal symmetry.

Among the β-Ti alloys, Niinomi *et al*. reported a new alloy, namely Ti-29Nb-13Ta-4.6Zr (mass%)^9^; which exhibits a lower Young’s modulus of ~65 GPa^10^. Thus, the Ti-29Nb-13Ta-4.6Zr alloy is considered a novel class of implant material that has undergone significant development^9^,^10^,^11^,^12^. However, its Young’s modulus remains higher than that of human bone, and so further research is still required. The control of the implant’s inner structure, for example the introduction of a porous structure, is considered as one of the approaches for the further reduction of the Young’s modulus of the implant^13^. However, there still exists an argument to use a porous implant for some applications where a large load is applied (e.g., artificial hip joints) in terms of the reliability of its mechanical properties.

Our group proposed a novel approach for lowering the Young’s modulus of the material itself, namely the use of the single crystal. The Young’s modulus in crystals is known to show orientation dependence even in the bcc-structured crystal exhibiting high crystal symmetry, and so it may be possible to reduce the Young’s modulus in the implant using the single crystal. In order to validate this concept, we examined the anisotropy of the elastic properties of the Ti-29Nb-13Ta-4.6Zr alloy using the single crystal^14^,^15^. A strong orientation dependence of the Young’s modulus was indeed confirmed, with the Young’s modulus exhibiting its highest value (~80 GPa) along the <111> orientation, and its lowest value along the <001> orientation (~35 GPa). The lowest Young’s modulus along the <001> orientation is close to that of cortical human bones (~10–30 GPa). These results imply the feasibility of using ‘single crystalline β-Ti implant’ as new hard tissue replacements for suppressing the stress shielding by the implant.

For the development of the ‘single crystalline implant’, not only the elastic properties but also the control of mechanical properties is important. For example, strength and resistance to fracture and fatigue should be sufficiently high. Studies have shown that the plastic deformation behavior also shows an orientation dependence in β-Ti alloys^16^,^17^,^18^. Although many reports exist on the mechanical properties of the Ti-29Nb-13Ta-4.6Zr alloy in a polycrystalline form^9^,^10^,^11^,^12^, our preliminary single crystal report appears to be the only one published to date^19^. Thus, the orientation dependence of the plastic deformation behavior of Ti-29Nb-13Ta-4.6Zr has not yet been fully investigated. In addition, the mechanical properties of β-Ti alloys vary significantly with heat treatment^2^,^18^. This has also been reported for the Ti-29Nb-13Ta-4.6Zr alloy^20^, although details were not sufficiently elucidated yet. We, therefore, chose to investigate the plastic deformation behavior of a Ti-29Nb-13Ta-4.6Zr biomedical implant alloy single crystal, and focusing on its orientation dependence and the influence of microstructure. From the results, the possibility of the development of the ‘single crystalline β-Ti implant’ by using the Ti-29Nb-13Ta-4.6Zr alloy single crystal was discussed.

## Results

### Relation between the microstructure and mechanical properties of the Ti-29Nb-13Ta-4.6Zr alloy single crystal

**Figure 1:**
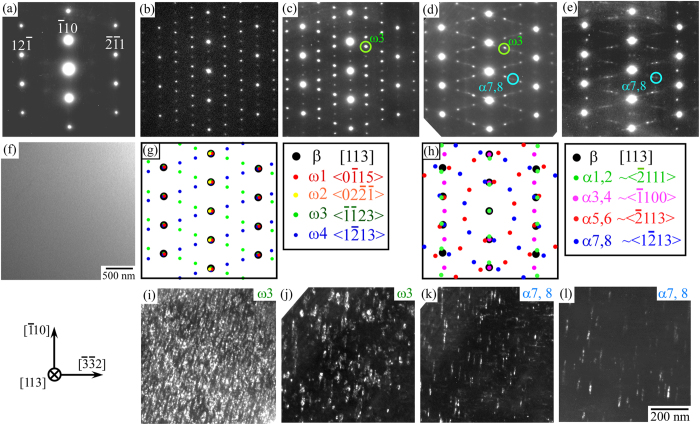
Variations in microstructure with heat treatment examined by TEM. (**a–e**) SAED patterns of the Ti-29Nb-13Ta-4.6Zr single crystal observed along the [113] direction, in specimens of (**a**) as-ST specimen, and annealed at (**b**) 573 K, (**c**) 598 K, (**d**) 673 K, and (**e**) 723 K for 259.2 ks, respectively. (**f**) Bright-field image of the as-ST specimen. (**g,h**) Simulated diffraction patterns derived from (**g**) ω-phase and (**h**) α-phase precipitates with distinct orientation relationships with respect to the β-matrix phase. (**i–l**) Dark-field images of the (**i**) precipitated ω-phases in the specimen annealed at 598 K for 259.2 ks, (**j,k**) precipitated ω-phases and α-phases in the specimen annealed at 673 K for 259.2 ks, and (**l**) precipitated α-phases in the specimen annealed at 723 K for 259.2 ks. The reflection spots observed for dark-field images are indicated as circles in Fig. 1(c–e).

To control the mechanical properties of the β-Ti alloys, control of the microstructure by heat treatment is useful. To achieve this, variations in the microstructure depending on the heat treatment conditions were first examined in the Ti-29Nb-13Ta-4.6Zr single crystal. Figure 1(a–e) show the variation in the selected area electron diffraction (SAED) patterns observed along the [113] direction upon heat treatment, in the original as- solution-treated (ST) single-crystalline specimen and those annealed at 573, 598, 673, and 723 K for 259.2 ks. As shown in Fig. 1(a), only the diffraction spots derived from the β-phase with a bcc structure were clearly observed in the ST specimen, demonstrating that the ST single crystal was composed of a β-single-phase, as is also confirmed by the bright field image shown in Fig. 1(f). In contrast, a number of additional spots were observed in the annealed specimens, as shown in Fig. 1(b–e). The positions and intensities of the extra spots varied with annealing temperature, suggesting that different phases precipitated at different temperatures. The precipitates present in the β-matrix-phase are supposed to be either a ω-phase with a hexagonal unit cell, or an α-phase with a hexagonal close-packed (hcp) unit cell. It is generally known that these phases precipitate with distinct relationships to the β-Ti matrix phase as follows:


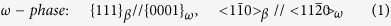



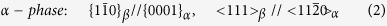


According to these relationships, four equivalent variants are considered for the ω-precipitates, and 12 variants are considered for the α-precipitates (see Supplementary Table S1). Taking their crystallographic orientations into consideration, Fig. 1(g,h) show the calculated SAED patterns from the precipitated ω- and α-phase variants in the β-matrix-phase when observed along [113]_β_. As only eight net diffraction patterns with relatively low lattice indices among the 12 variants were indicated for the simulated α-phase diffraction pattern in Fig. 1(h), the observed TEM diffraction patterns could not be perfectly reproduced. However, by comparing to the experimental SAED patterns, it was elucidated that the specimens were composed of a β + ω two-phase at 573 K and 598 K, a β + ω + α three-phase at 673 K, and a β + α two-phase at 723 K. This was further confirmed by dark-field observations. Figure 1(i) show the dark field images observed in the specimen annealed at 598 K, with the circled spots shown in Fig. 1(c). Using the spot, the ω-phases were confirmed to be densely precipitated in the specimen. The ω-phases exhibited ellipse-like shapes, having the long axis aligned along the [0001] direction, with an average length of ~10 nm. Figure 1(j,k) show the dark-field images of the specimen annealed at 673 K. With the same spot used for observation in Fig. 1(i), the presence of ω-precipitates was confirmed as shown in Fig. 1(j). In addition, using the spot named “α7,8” in Fig. 1(d), the precipitation of the α-phase was also observed as shown in Fig. 1(k). The α-phases precipitated abundantly with thin plate-like shapes. In the specimen annealed at 723 K, α-phase precipitates were confirmed to be slightly coarser, but maintained their plate-like shape, as shown in Fig. 1(l).

**Figure 2:**
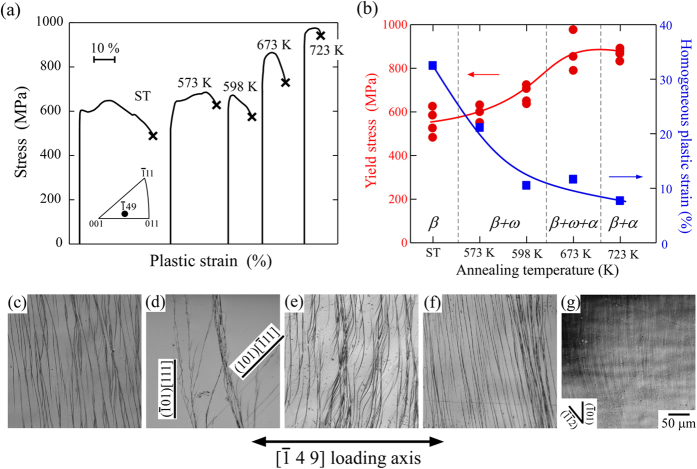
Variations in deformation behaviors with heat treatment. (**a**) Variations in typical stress-strain curves of the Ti-29Nb-13Ta-4.6Zr single crystals deformed at [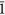
49] orientation, with heat treatment. (**b**) Corresponding variations in yield stress (0.2% offset stress) and homogeneous plastic strain as a function of the annealing temperature. (**c–g**) Optical micrographs showing the variations in morphology of the slip traces with heat treatment, introduced in the specimens deformed at [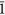
49] loading orientation to ~2% plastic strain. (**c**) as-ST specimen, and the specimens annealing at (**d**) 573 K, (**e**) 598 K, (**f**) 673 K, and (**g**) 723 K, respectively. The surface planes of the observed specimens are parallel to (11 5 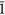).

The change in mechanical properties of the Ti-29Nb-13Ta-4.6Zr alloy single crystal following heat treatment was examined by compression tests at a [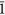
49] loading orientation, where the Schmid factor for (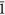
01)[111] is 0.500, as shown in Table 1. Figure 2(a) shows the typical stress-strain curves of the specimens deformed at a [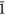
49] loading orientation. Furthermore, Fig. 2(b) shows the variations in yield stress and homogeneous plastic strain before fracture, both as a function of annealing temperature. The yield stress monotonously increased with increasing annealing temperature, while the strengthening behavior was largely divided into two temperature regions. Between 573 and 598 K, where only the ω-phase precipitated, alloy strengthening was relatively small, approximately less than 150 MPa. However, at increased temperatures (673–723 K) where the α-phase also precipitated, the stress increase was pronounced, to more than 300 MPa. Accompanying the increase in yield stress, the fracture strain decreased rapidly with rising annealing temperature. Focusing on the shape of the stress-strain curve, the ST specimen exhibited no work hardening after yielding. The flow stress showed a gradual decrease as deformation proceeded, but over 30% plastic strain could be obtained before fracture. With an increase in the annealing temperature, the steady flow stress region after yielding was shorter, and thus, the fracture strain decreased. In the specimens annealed above 598 K, where a large amount of α-phase was precipitated, the fracture strain was decreased lower than 10%.

Figure 2(c–g) shows the morphology of the deformation markings introduced in the specimens by deformation at a [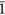
49] loading orientation to ~2% plastic strain, observed on the (11 5 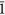) surface. The features of the deformation traces suggest that the dislocation slip carried the strain in deformation, and other deformation modes were not observed irrespective of the heat treatment condition. By the two-face slip trace analysis, the slip plane of the dislocations was confirmed to be macroscopically parallel to (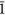
01). From the consideration based on the Schmid factor shown in Table 1, the primary operative slip system was expected to be (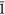
01) [111]. This was confirmed by the transmission electron microscopy (TEM) observation, as described later. In addition, the (101)[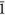
11] slip was locally observed as a secondary slip system. It should be noted that although the traces of primary slip were aligned macroscopically parallel to (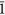
01), they were not straight. The slip traces exhibited a wave-like form in all specimens. This suggests that the [111] dislocations frequently cross-slip from the (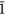
01) plane to other planes.

Although the (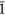
01)[111] slip was confirmed in all specimens, the detailed morphology of the slip traces varied under different heat treatment conditions. In the ST specimen, slip traces were relatively homogeneously distributed over the specimen surface. However, in the specimens annealed at 573 K and 598 K, the slip traces were coarsely introduced. The contrast of the traces became stronger with the concentration of slip traces, resulting in the formation of slip bands. Between the strong slip bands, no fine slip trace was observed. The formation of microcracks was occasionally observed along the slip bands, leading to a decrease in specimen ductility. Interestingly, at annealing temperatures above 673 K, the slip traces were once again introduced homogeneously, and the contrast became weak. In the specimen annealed at 723 K the slip traces were too faint to accurately determine the slip plane.

**Figure 3:**
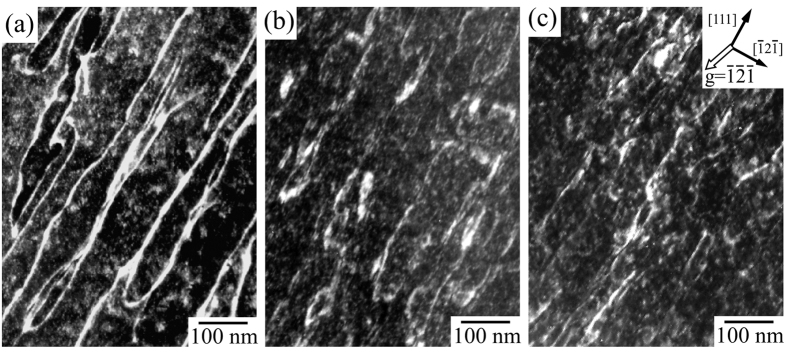
Dislocation structure in specimens deformed to ~2% plastic strain at [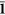
49] loading orientation. (**a**) As-ST specimen, (**b**) annealed at 573 K, and (**c**) annealed at 598 K. Foil//(10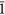) slip plane, and the observed beam direction (B)//[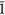
01].

The dislocation structures introduced in the specimens were examined by TEM. Figure 3(a) shows the dislocation structure of the ST specimen composed of a β-single phase, observed on the (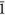
01) slip plane. Relatively straight morphologies of the dislocations were abundantly observed. By g·b contrast analysis, the Burgers vector of the dislocations was confirmed to be parallel to [111]. The crystal orientations examined by SAED pattern analysis are indicated in the figures. They show that the aligned directions of the dislocations were almost parallel to [111], indicating that the observed dislocations are predominantly screw dislocations. Figure 3(b,c) show the dislocations observed in the 573 K and 598 K annealed specimens, respectively, where ω-phases precipitated in the β-matrix phase. As the same as that observed in the ST specimen, the Burgers vectors of the dislocations were parallel to [111], and they predominantly exhibited screw characteristics. However, in the 573 K and 598 K annealed specimens, the dislocation segments were shorter, and were curled in comparison to those in the ST specimen. This variation demonstrates that precipitation of the ω-phase significantly affected the motion of the [111] dislocation. TEM observation was also conducted in the deformed specimen annealed at 673 K and 723 K. In those specimens, however, the dislocation structure could not be sufficiently examined since the images of the dislocations were faint owing to the presence of large amounts of α-phases as shown in Fig. 1(k,l). In those specimens the slip traces became anomalously faint, as shown in Fig. 2(f,g).

### Orientation dependence of the plastic deformation behavior

**Figure 4:**
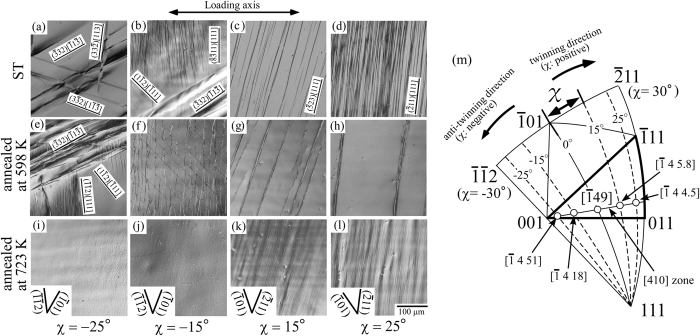
Variation in the morphology of deformation markings as a function of loading orientation in the specimens deformed to ~2% plastic strain. (**a–d**) As-ST specimens. (**e–h**) Specimens annealed at 598 K. (**i–l**) Specimens annealed at 723 K. The loading orientations were (**a,e,i**) 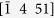 (χ = −25°), (**b,f,j**) 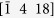 (χ = −15°), (**c,g,k**) 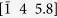 (χ = 15°), and (**d,h,l**) 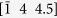 (χ = 25°), respectively. (**m**) Stereographic projection of the selected loading axes for the compression tests.

To examine the orientation dependence of the plastic deformation behavior of Ti-29Nb-13Ta-4.6Zr alloy, specimens with five different loading axes, including the 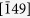 orientation, were prepared on the [410] zone axis in a [001]-[011]-[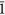
11] standard triangle, as shown in Fig. 4(m), and their plastic deformation behaviors were compared. The five loading axes chosen were parallel to 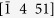, 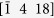, 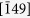, 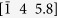, and 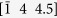. In the bcc-structured crystal, the angle between the maximum resolved shear stress plane (MRSSP) in the [111] zone and the reference 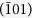 plane, defined as “χ” is important in governing the orientation dependence of the plastic deformation behavior. In this aspect, the loading axes selected in this study correspond to χ = −25° at 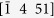, χ = −15° at 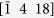, χ = 0° at 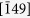, χ = +15° at 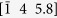, and χ = +25° at 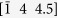 as shown in Fig. 4(m).

**Figure 5:**
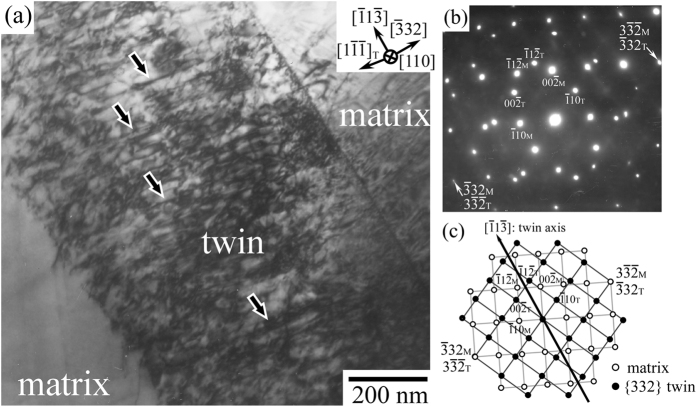
Observation of {332} twin by TEM. (**a**) Bright-field TEM image observed in the as-ST specimen deformed at 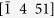 (χ = −25°) to ~2% plastic strain. Observed beam direction is parallel to [110]. (**b**) SAED pattern obtained at the boundary of the band-like deformation product. (**c**) Corresponding key diagram. Analysis of the SAED pattern demonstrates that the band-like deformation product is the (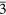
32)[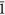
1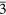] deformation twin.

Figure 4(a–l) show the variations in the deformation markings with loading orientation in the ST specimens and those annealed at 598 K and 723 K after ~2% deformation. Two notable variations in the deformation marking morphology were observed, varying with heat treatment condition and loading orientation. These variations are the change in the operative deformation mode and the change in slip plane of the [111] dislocation. In terms of the operative deformation mode, in the ST specimen deformed at 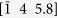 (χ = 15°), large amounts of slip traces were introduced comparable to 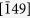 (χ = 0°), as shown in Fig. 4(c). Comparable observations were also made in the specimen deformed at 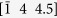 (χ = 25°, Fig. 4(d)), although a small amount of band-like deformation product was observed at the ends of the specimen. In contrast, in the specimens deformed at 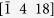 (χ = −15°), coarse band-like deformation products were formed in addition to the slip traces (Fig. 4(b)), and in the 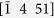 (χ = −25°) specimen the band-like deformation products became the predominant deformation mode (Fig. 4(a)). To clarify the origin of the band-like deformation products, TEM observation was conducted on the specimen deformed at 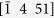 (χ = −25°). Figure 5(a) shows the bright-field image of the band-like deformation product observed along [110], and Fig. 5(b) shows the corresponding SAED pattern obtained at its boundary. The SAED pattern demonstrates that the deformation band shows a twin orientation relationship with the interface parallel to {332} with respect to the matrix, as the corresponding key diagram is shown in Fig. 5(c). The result demonstrates that the formation of the {332} twin in the Ti-29Nb-13Ta-4.6Zr crystal varying with loading orientation.

As shown in Table 1, 12 kinds of {332} twinning systems were considered. By the two-face trace analysis of the twin interfaces using an optical microscope, formation of the (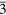
32) twin was most predominantly confirmed among them. In addition, some amount of (3
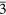
2) twin and small amounts of the (332) and (33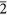) twins were also observed in the ST specimens with loading orientations at 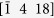 and 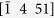. The formation of {332} twins was also observed in the specimen annealed at 598 K in the deformation at 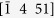 (χ = −25°). However, in this annealed specimen the formation of {332} twins was not observed at 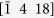 (χ = −15°), and instead, slip was observed, differing from that in the ST specimen. Furthermore, in the specimen annealed at 723 K, the formation of the {332} twins was suppressed, and only the slip traces were observed at all examined loading orientations, although the contrast of the slip traces was faint, as shown in Fig. 4(i–l). These results indicate that the formation of {332} twins is hindered by ω-phase precipitation, and the extent of suppression was more significant with the precipitation of α-phase.

Another notable point in the deformed specimens was that the slip plane in the deformed specimens varied depending on the loading axis. As indicated in Fig. 2(c–g), the slip plane of the predominant slip was nearly parallel to the (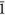
01) at 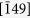 orientation (χ = 0°). However, the slip plane deviated from (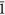
01) at other loading orientations. In the ST specimens, slip planes of the predominant dislocations with the Burgers vector parallel to [111] were examined by a two-face slip analysis at the central position of the specimen, and were determined to be close to 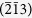, 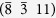, 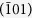, 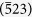, and 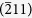 at the 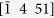, 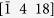, 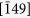, 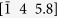, and 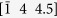 loading orientations, respectively, as shown in Figs 2(c) and 4(a–d). Analysis was also conducted on the specimens annealed at 598 K and 723 K, showing that comparable transitions occurred in the slip plane depending on the loading orientation, irrespective of the heat-treatment condition. However, in the specimen annealed at 723 K, the slip planes could not be determined precisely at the 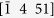 and 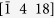 loading orientations because of the faint slip traces.

**Figure 6:**
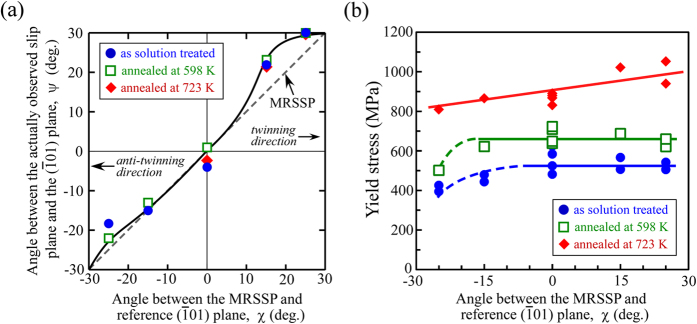
Orientation dependence of the deformation behavior. (**a**) Variations in the slip plane of [111] dislocations with loading orientation; the so-called ψ−χ curve measured in the Ti-29Nb-13Ta-4.6Zr single crystal compressed at RT. (**b**) Corresponding orientation dependence of the yield stress examined.

In order to discuss quantitatively the variations in the slip plane, ψ−χ curve analysis was conducted, and the results are summarized in Fig. 6(a). The χ is defined as the angle between the MRSSP in the [111] zone and the reference 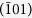 plane, which varies depending on the loading axis, as shown in Fig. 4(m). Furthermore, ψ is the angle between the actually observed slip plane and the 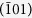 plane. If the slip plane does not vary from (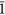
01) in any loading orientation, a horizontal line can be drawn along ψ = 0, whereas if the slip plane freely varies depending on the resolved shear stress, it will vary along the dashed line shown in Fig. 6(a), which corresponds to the MRSSP (i.e., ψ = χ). It was found experimentally that the slip plane generally varied along the MRSSP, although a selection of slip traces showed a slight deviation from the MRSSP at χ ≥ 15°. The observed slip plane moved from the MRSSP to 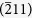 at χ ≥ 15°.

Figure 6(b) shows the variation in yield stress with the loading axis defined by the χ-value, focused for five loading orientations on the [410] zone axis. As shown in Fig. 2(b), the yield stress increased with annealing, with a larger increase upon annealing at 723 K compared to 598 K at the 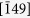 orientation (χ = 0°). Similar observation were made at all loading orientations. It should be noted that the yield stress gave comparable values at all loading orientations in the ST specimens, i.e., the yield stress showed little orientation dependence, except for that at χ = −25°. At around χ = −25°, however, the yield stress slightly decreased compared to other loading orientations. Almost similar orientation dependence of the yield stress was observed in the specimens annealed at 598 K. Note that the loading orientations where the slight decreases in yield stress were observed correspond to the regions where {332} twins formed in compression tests, as shown in Fig. 4. On the other hand, the yield stress of the specimens annealed at 723 K show a slight decrease as decreasing in the χ-value, although the formation of deformation twin was not observed in them.

## Discussion

We found that the plastic deformation behavior of the solution-treated Ti-29Nb-13Ta-4.6Zr alloy single crystal was considerably isotropic and the orientation dependence of the yield stress was small, particularly in the loading orientations where <111> dislocations are operative, as shown in Fig. 6(b). This differed from the behavior of the Ti-15Mo-5Zr-3Al β-phase single crystal, which is another promising material for use in biomedical implant applications, as approved by the International Organization for Standardization (ISO). As previously reported^21^, the yield stress of the Ti-15Mo-5Zr-3Al single crystal showed a relatively strong orientation dependence, increasing with a decrease in χ. Thus, the compressive yield stress is lower at the loading orientation where the 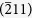
[111] slip is preferentially operative (the twinning sense direction) than where the 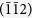
[111] slip is preferential (anti-twinning sense direction). The compressive yield stress at χ = −25° was approximately 1.2 times higher than that at χ = 25° in Ti-15Mo-5Zr-3Al. In contrast, the compressive yield stress in Ti-29Nb-13Ta-4.6Zr was comparable at all orientations except for those at approximately χ = −25°, where the formation of {332} twins occurred. These different features of the plastic deformation behavior in the bcc-structured β-Ti crystals must be derived from the variation in the non-planar dislocation core structure of the <111> dislocation, as discussed previously^21^. In Ti-15Mo-5Zr-3Al, the <111> dislocation may exhibit a more complicated three-dimensional dislocation core structure that induces the twinning/anti-twinning sense asymmetry in the dislocation motion when compared to Ti-29Nb-13Ta-4.6Zr. Indeed, in Ti-15Mo-5Zr-3Al, the transition behavior of the slip plane largely deviated from the MRSSP towards 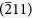
21. We proposed that such variations in the plastic deformation behavior of multi-component β-Ti alloys are predominantly affected by the properties of the major bcc-phase stabilizing constituent element itself, in this case Nb and Mo, respectively. This hypothesis is derived from the studies of Duesbery and Vitek^22^. They studied the plastic deformation behavior of various pure bcc metals, and found that plastic anisotropy is more stronger in group VIB metals (Mo and W) than in group VB metals (Nb and Ta)^22^. This is in agreement with the behavior of the Ti-15Mo-5Zr-3Al and Ti-29Nb-13Ta-4.6Zr systems. In addition, Hanada reported that the plastic deformation behavior of a Ti-Nb binary single crystal (Ti-52wt.%Nb) showed little orientation dependence at 300 K^18^, a property also observed in our Ti-29Nb-13Ta-4.6Zr crystal. However, Hanada also reported that the Ti-Nb binary single crystal exhibits strong anisotropic deformation behavior when the deformation temperature was decreased to 77 K. This suggests that the anisotropic feature of the plastic behavior in the β-Ti crystal is controlled by both the alloy composition and the deformation temperature. The effect of temperature can be explained by the temperature dependence of the thermal activation process for the transformation of the three-dimensional non-planar dislocation core structure to the mobile planar structure. Further studies using single crystals are required to clarify details regarding the controlling factor of the plastic deformation behavior of multi-element β-Ti alloys.

As described above, the yield stress of the solution-treated Ti-29Nb-13Ta-4.6Zr crystal shows little orientation dependence in the wide loading orientation region where the operation of <111> dislocations are dominant. However, in the ST specimen the yield stress slightly decreased at around −25° ≤ χ ≤ −15°. In the specimens exhibiting lower yield stress, the formation of {332} twins was observed, as shown in Fig. 4(a,b). The slight decrease in yield stress at approximately χ = −25° must, therefore, be ascribed to the operation of the {332} twins. In the deformed specimen at 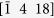 (χ = −15°) and 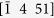 (χ = −25°) loading orientations, the formation of (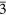
32), (3
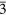
2), (332), and (33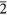) twins were confirmed among the twelve {332} twinning systems. As shown in Table 1, when taking into consideration the tension-compression asymmetry (polarization) on the formation of twins, the four twins can be formed under compression, while the others cannot be formed without tensile stress at those loading orientations. This result demonstrates that the {332} twins formation tendencies can be estimated roughly by focusing on the asymmetry of twinning shear that can be evaluated from considering the conventional Schmid factor. However, the discrepancy expected from the consideration based on the Schmid factor was also monitored. The formation of {332} twins is limited at a loading orientation of around −25° ≤ χ ≤ −15° in the ST specimen, and their formation was not observed in specimens with a loading axis of χ > −15°, despite a small amount of twins being observed in the specimen with 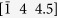 loading orientation (χ = 25°). The largest Schmid factor for {332} twins among the twelve systems was 0.425^c^ at 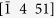 (χ = −25°) and 0.472^c^ at 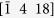 (χ = −15°) loading orientations, as shown in Table 1. It is to note, however, that at other loading orientations at χ > −15°, some {332} twins maintained relatively high Schmid factors; 0.476^c^ at 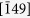 (χ = 0°), 0.420^c^ at 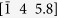 (χ = 15°), and 0.354^c^ at 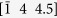 (χ = 25°). As described above, the yield stress of the Ti-29Nb-13Ta-4.6Zr crystal showed little orientation dependence. As the slip plane of the <111> dislocation varied nearly along the MRRSP, the Schmid factor for the <111> slip must maintain high values of ~0.48–0.50 at all loading orientations. This implies that the critical resolved shear stress (CRSS) for the <111> slip varies very little with the loading orientation. Thus, under the Schmid factor-derived consideration, the formation of {332} twins was expected at all investigated loading orientations. However, this differed from the experimental results, indicating that the CRSS for the formation of {332} twins does not obey the Schmid law perfectly, and orientation dependence must be exhibited. Related assumptions have been reported previously by Hanada *et al*. in other alloy systems^16^,^17^,^18^.

Another notable point is that the differences in orientation dependence of yield stress with heat treatment conditions. As shown in Fig. 6(b), the orientation dependence of the yield stress was negligibly small in the specimens annealed at 598 K in which the ω-phase was precipitated, as similarly to that in the ST specimens, at the loading orientations where <111> dislocations were operative. On the other hand, the yield stress of the specimens annealed at 723 K in which the α-phase was precipitated tended to show a slight decrease as decreasing in the χ-value; i.e. the isotropic plasticity degenerated with the presence of α-phase. In the specimens annealed at 723 K, the motion of dislocation was confirmed to control the deformation behavior at all loading orientations, but the variation in the orientation dependence of the yield stress exhibited the “inverse” trend compared to those observed in conventional bcc-metals as described above. As shown in Figs 2 and 4, the morphology of the slip traces in the specimens annealed at 723 K was also largely different from those in the ST specimens and those annealed at 598 K. These results imply that the interacting behaviors of <111> dislocations with ω- and α-precipitates are different, and a different deformation mechanism will exist and control the mechanical properties of the specimens in which α-phases were precipitated, compared to those in the ST specimens and ω-phase precipitated specimens. Further experimental data are required to discuss such mechanisms, and studies are ongoing in our group.

It was previously reported that the operative deformation mode in the β-Ti alloy varies depending on the β-phase stability governed by the alloy composition and heat-treatment condition^17^,^18^,^23^. In the alloy exhibiting low β-phase stability, the {332} twins tend to be operative. The lattice modulation plays a key role in facilitating the formation of the {332} twin, and it is enhanced in unstable β-Ti alloys^24^. While in β-Ti alloys with high β-phase stability, the <111> slip operation became the dominant deformation mode. The present study clarified that the dominant operative deformation mode varied with loading orientation in the Ti-29Nb-13Ta-4.6Zr alloy single crystals, different from the behavior in Ti-15Mo-5Zr-3Al single crystal^21^. This indicated that β-phase stability in this alloy is the intermediate where the operative deformation mode changes. It was previously reported that the {332} twin operation is beneficial for increasing alloy elongation, although it accompanies a reduction in yield stress, and contradictory properties result from slip operations, i.e., an increase in yield stress accompanied by a reduction in ductility^25^. Thus, the simultaneous twin and slip operations in polycrystalline alloys must be beneficial to ensure both the strength and ductility of the alloy, as pointed out by Min *et al*.^26^. Furthermore, the increase in the number of operative deformation modes is beneficial for increasing the deformability of the polycrystalline alloy by ensuring that the Mises criterion is met. This accounts for the Ti-29Nb-13Ta-4.6Zr alloy exhibiting good mechanical properties with superior strength and ductility.

In conclusion, the plastic deformation behavior of Ti-29Nb-13Ta-4.6Zr alloy single crystal was first clarified in this study. The <111> slip and {332} twin were identified to be the operative deformation modes. Their operations showed strong crystal orientation dependence, but the resultant variation in yield stress was not significant. That is, the Ti-29Nb-13Ta-4.6Zr alloy single crystal was found to show relatively isotropic plastic properties, which is contradict to its strongly anisotropic elastic properties. This “plastically almost-isotropic and elastically highly-anisotropic” nature is desirable for the development of ‘single crystalline β-Ti implant’ as new hard tissue replacements for suppressing the stress shielding while maintaining the high mechanical reliability.

## Method

### Fabrication of single crystalline specimens

A mother alloy with a composition of Ti-29Nb-13Ta-4.6Zr (mass%) was supplied by Daido Steel Co. Ltd., Japan. It was prepared by vacuum arc remelting and subsequent forging. From the ingot, single crystals were grown in a furnace (SCI-MDH 20020, Canon machinery, Japan) using the optical floating zone (FZ) method, with a crystal growth rate of 2.5 mm/h, under a flow of high purity Ar gas. In order to ensure the homogeneity of the single crystal composition, the mother ingot and the obtained single crystals were rotated (6 rpm) at opposite directions to each other during crystal growth. Following the crystal growth process, the single crystal was slowly cooled to room temperature (RT, 293 K) over 40 h.

The chemical compositions of the mother ingot and the single crystal were determined by inductively coupled plasma (ICP) analysis and by the inert gas fusion method. As a result, it was confirmed that the chemical composition of the obtained single crystal was confirmed to be comparable to that of the mother alloy for all constituent elements in the quaternary system, with low O concentration (see Supplementary Table S2). The obtained single crystal was subjected to solution treatment (ST) at 1063 K for 3.6 ks under Ar atmosphere, followed by water quenching (WQ). Some crystals were encapsulated once again and annealed at varying temperatures (573, 598, 673, and 723 K) for 259.2 ks, followed by WQ, to provide microstructural variation in the β-phase single crystals. The constituent phase was identified by transmission electron microscopy (TEM, JEM-3010, JEOL, Japan).

### Examination of mechanical properties

Compression tests were performed to examine the mechanical properties at RT. Rectangular specimens (2.0 × 2.0 × 5.0 mm^3^) were cut by electric discharge machining. After mechanical polishing with SiC paper (#400-2000), the specimens were electropolished in a perchloric acid/butanol/methanol solution 6/35/59 (vol.%). Single crystal orientation was determined using the X-ray back Laue diffraction method with an accuracy of 1°. The loading axis orientation of the compressive specimens was mainly chosen to be [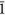
49] where the Schmid factor of the (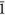
01)[111] primary slip system had a maximum value of 0.500. Other specimens with four different loading axes were also prepared in a [001]-[011]-[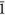
11] standard stereographic triangle as shown in Fig. 4(m), to examine the orientation dependence of the plastic behavior.

Compression tests were conducted on an Instron-type mechanical testing machine (Autograph AG-5000C, Shimadzu, Japan) at a nominal strain rate of 1.67 × 10^−4^ s^−1^ under vacuum at RT. Slip markings introduced in the deformed specimens were observed using an optical microscope (OM, BX60M, Olympus, Japan) equipped with Nomarski interference contrast. The slip plane of the dislocations was determined by the two-face slip trace analysis using the OM, and its transition depending on the loading orientation was quantitatively analyzed by the so-called ψ−χ curve method. Deformation structures in the compressed specimens were observed in a TEM operated at 300 kV.

## Additional Information

**How to cite this article**: Hagihara, K. *et al*. Isotropic plasticity of β-type Ti-29Nb-13Ta-4.6Zr alloy single crystals for the development of single crystalline β-Ti implants. *Sci. Rep.* **6**, 29779; doi: 10.1038/srep29779 (2016).

## Supplementary Material

Supplementary Information

## Figures and Tables

**Table 1:** Schmid factors for possible deformation modes at the five examined loading orientations.

Deformation mode	Slip (twinning) system	Schmid factors				
		[ 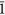 4 51] (χ = −25°)	[ 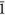 4 18] (χ = −15°)	[ 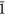 4 9] (χ = 0°)	[ 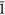 4 5.8] (χ = 15°)	[ 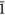 4 4.5] (χ = 25°)
{110} <111> slip	( 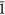 0 1) [1 1 1]	0.438	0.479	0.500	0.483	0.453
	(1 0 1) [ 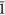 1 1]	0.437	0.469	0.467	0.419	0.366
	( 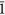 0 1) [1 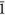 1]	0.373	0.293	0.167	0.045	0.028
	(1 0 1) [1 1 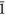 ]	0.374	0.303	0.200	0.109	0.059
	( 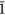 1 0) [1 1 1]	0.042	0.128	0.250	0.353	0.410
	(1 1 0) [ 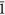 1 1]	0.026	0.084	0.175	0.260	0.311
	(1 1 0) [1 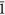 1]	0.022	0.047	0.050	0.020	0.015
	( 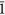 1 0) [1 1 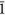 ]	0.037	0.091	0.125	0.113	0.083
	(0 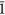 1) [1 1 1]	0.396	0.350	0.250	0.129	0.043
	(0 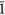 1) [ 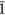 1 1]	0.410	0.384	0.292	0.159	0.055
	(0 1 1) [1 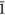 1]	0.395	0.340	0.217	0.065	0.044
	(0 1 1) [1 1 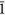 ]	0.412	0.394	0.325	0.223	0.142
{112} <111> slip	(1 1 2) [ 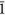 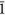 1]	0.454	0.403	0.303	0.192	0.116
	( 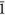 1 2) [1 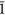 1]	0.443	0.366	0.221	0.064	0.042
	(1 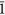 2) [ 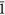 1 1]	0.489	0.492	0.438	0.333	0.243
	(1 1 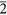 ) [ 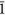 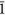 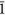 ]	0.481	0.479	0.433	0.354	0.289
	(1 2 1) [ 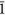 1 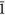 ]	0.240	0.224	0.154	0.049	0.034
	( 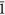 2 1) [1 1 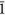 ]	0.259	0.280	0.260	0.194	0.130
	(1 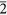 1) [ 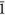 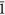 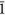 ]	0.204	0.128	0.000	0.129	0.211
	(1 2 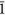 ) [ 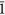 1 1]	0.222	0.173	0.067	0.059	0.148
	(2 1 1) [1 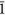 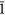 ]	0.267	0.319	0.370	0.392	0.391
	( 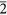 1 1) [ 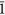 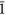 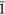 ]	0.277	0.350	0.433	0.483	0.498
	(2 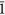 1) [1 1 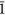 ]	0.194	0.122	0.043	0.002	0.014
	(2 1 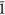 ) [1 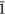 1]	0.203	0.142	0.067	0.015	0.007
{332} <113> twin	(3 3 2) [1 1 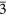 ]	0.409^c^	0.433^c^	0.425^c^	0.377^c^	0.328^c^
	( 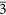 3 2) [ 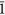 1 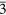 ]	0.425^c^	0.472^c^	0.476^c^	0.420^c^	0.354^c^
	(3 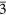 2) [1 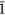 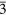 ]	0.338^c^	0.230^c^	0.063^c^	0.095^t^	0.189^t^
	( 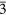 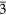 2) [ 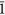 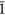 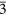 ]	0.356^c^	0.288^c^	0.177^c^	0.069^c^	0.002^c^
	(3 2 3) [1 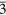 1]	0.147^t^	0.053^t^	0.084^c^	0.203^c^	0.270^c^
	( 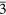 2 3) [ 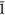 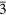 1]	0.161^t^	0.083^t^	0.050^c^	0.186^c^	0.273^c^
	(3 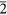 3) [1 1 3]	0.216^t^	0.235^t^	0.210^t^	0.138^t^	0.069^t^
	( 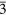 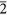 3) [ 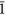 3 1]	0.233^t^	0.288^t^	0.317^t^	0.297^t^	0.258^t^
	(2 3 3) [ 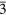 1 1]	0.232^t^	0.304^t^	0.388^t^	0.445^t^	0.466^t^
	( 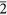 3 3) [1 1 3]	0.213^t^	0.244^t^	0.269^t^	0.271^t^	0.261^t^
	(2 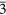 3) [ 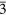 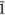 1]	0.171^t^	0.127^t^	0.068^t^	0.021^t^	0.002^t^
	( 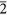 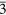 3) [3 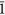 1]	0.154^t^	0.090^t^	0.022^t^	0.011^c^	0.015^c^

The superscript t and c indicate the deformation twins operative in the tensile and compressive deformation, respectively.
