# Erratum: Isotropic plasticity of β-type Ti-29Nb-13Ta-4.6Zr alloy single crystals for the development of single crystalline β-Ti implants

**DOI:** 10.1038/srep31681

**Published:** 2016-08-16

**Authors:** Koji Hagihara, Takayoshi Nakano, Hideaki Maki, Yukichi Umakoshi, Mitsuo Niinomi

Scientific Reports 6: Article number: 2977910.1038/srep29779; published online: 07 15 2016; updated: 08 16 2016

This Article contains typographical errors in Table 1. In the column ‘Slip (twinning) system’, the seventh {332} <113> twin value “(3 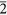
3) [1 3 1]” is incorrectly given as “(3 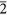
3) [1 1 3]”. In addition, the tenth {332} <113> twin value “(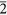 3 3) [3 1 1]” is incorrectly given as “(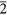 3 3) [1 1 3]”.

