# Supplementary material for: Isotropic plasticity of β-type Ti-29Nb-13Ta-4.6Zr alloy single crystals for the development of single crystalline β-Ti implants
Source: Sci Rep. 2016 Jul 15;6:29779. doi: 10.1038/srep29779 (PMC4945923; doi:10.1038/srep29779)
Supplement: Supplementary Information [file srep29779-s1.pdf]

SUPPLEMENTARY INFORMATION
for

Isotropic plasticity of β-type Ti-29Nb-13Ta-4.6Zr alloy single crystals for the development of single crystalline β-Ti implants

Koji Hagihara[1], Takayoshi Nakano[2*], Hideaki Maki[2],
Yukichi Umakoshi[2] and Mitsuo Niinomi[3]

[1] Department of Adaptive Machine Systems, Graduate School of Engineering, Osaka University, 2-1 Yamadaoka, Suita, Osaka 565-0871, Japan
[2] Division of Materials and Manufacturing Science, Graduate School of Engineering, Osaka University, 2-1 Yamadaoka, Suita, Osaka 565-0871, Japan
[3] Institute for Materials Research, Tohoku University, 2-1-1 Aoba-ku, Sendai, Miyagi 980-8577, Japan
* Corresponding author: nakano@mat.eng.osaka-u.ac.jp

Supplementary Table S1 | Crystal orientation relationships expected in precipitates. (a) Four possible ω-phase variants and (b) twelve α-phase variants precipitated in the β-phase single crystal with distinct orientation relationships described in the manuscript.

(a) ω-phase

ω1	$(111)\beta//(0001)\omega$	$[11\bar{2}]\beta//[01\bar{1}0]\omega$	$[1\bar{1}0]\beta//[2\bar{1}\bar{1}0]\omega$
ω2	$(11\bar{1})\beta//(0001)\omega$	$[112]\beta//[01\bar{1}0]\omega$	$[1\bar{1}0]\beta//[2\bar{1}\bar{1}0]\omega$
ω3	$(1\bar{1}1)\beta//(0001)\omega$	$[1\bar{1}\bar{2}]\beta//[01\bar{1}0]\omega$	$[110]\beta//[2\bar{1}\bar{1}0]\omega$
ω4	$(\bar{1}11)\beta//(0001)\omega$	$[\bar{1}1\bar{2}]\beta//[01\bar{1}0]\omega$	$[\bar{1}\bar{1}0]\beta//[2\bar{1}\bar{1}0]\omega$

(b) α-phase

α1	$(110)\beta//(0001)\alpha$	$[1\bar{1}2]\beta//[01\bar{1}0]\alpha$	$[\bar{1}11]\beta//[2\bar{1}\bar{1}0]\alpha$
α2	$(110)\beta//(0001)\alpha$	$[1\bar{1}\bar{2}]\beta//[01\bar{1}0]\alpha$	$[\bar{1}1\bar{1}]\beta//[2\bar{1}\bar{1}0]\alpha$
α3	$(1\bar{1}0)\beta//(0001)\alpha$	$[112]\beta//[01\bar{1}0]\alpha$	$[11\bar{1}]\beta//[2\bar{1}\bar{1}0]\alpha$
α4	$(1\bar{1}0)\beta//(0001)\alpha$	$[11\bar{2}]\beta//[01\bar{1}0]\alpha$	$[111]\beta//[2\bar{1}\bar{1}0]\alpha$
α5	$(101)\beta//(0001)\alpha$	$[12\bar{1}]\beta//[01\bar{1}0]\alpha$	$[1\bar{1}\bar{1}]\beta//[2\bar{1}\bar{1}0]\alpha$
α6	$(101)\beta//(0001)\alpha$	$[1\bar{2}\bar{1}]\beta//[01\bar{1}0]\alpha$	$[11\bar{1}]\beta//[2\bar{1}\bar{1}0]\alpha$
α7	$(011)\beta//(0001)\alpha$	$[2\bar{1}1]\beta//[01\bar{1}0]\alpha$	$[\bar{1}\bar{1}1]\beta//[2\bar{1}\bar{1}0]\alpha$
α8	$(011)\beta//(0001)\alpha$	$[\bar{2}\bar{1}1]\beta//[01\bar{1}0]\alpha$	$[1\bar{1}1]\beta//[2\bar{1}\bar{1}0]\alpha$
α9	$(\bar{1}01)\beta//(0001)\alpha$	$[121]\beta//[01\bar{1}0]\alpha$	$[1\bar{1}1]\beta//[2\bar{1}\bar{1}0]\alpha$
α10	$(\bar{1}01)\beta//(0001)\alpha$	$[1\bar{2}1]\beta//[01\bar{1}0]\alpha$	$[111]\beta//[2\bar{1}\bar{1}0]\alpha$
α11	$(0\bar{1}1)\beta//(0001)\alpha$	$[\bar{2}11]\beta//[01\bar{1}0]\alpha$	$[111]\beta//[2\bar{1}\bar{1}0]\alpha$
α12	$(0\bar{1}1)\beta//(0001)\alpha$	$[211]\beta//[01\bar{1}0]\alpha$	$[\bar{1}11]\beta//[2\bar{1}\bar{1}0]\alpha$

Supplementary Table S2 | Chemical compositions of the mother ingot and grown single crystal.

	Ti	Nb	Ta	Zr	O	N
Composition (wt.%)						
mother ingot	bal.	29.0	13.2	4.66	0.12	0.01
single crystal	bal.	29.2	13.5	4.54	0.17	0.03
Composition (at.%)						
mother ingot	bal.	20.1	4.7	3.3	0.48	0.05
single crystal	bal.	20.3	4.8	3.2	0.69	0.14
